# Annotations

**Published:** 1900-07-14

**Authors:** 


### Annotations.

### University Teaching in London.

Now that London has to all intents and purposes got its teaching University the question how and where, and by whom the medical teaching is to be done becomes urgent; and it must be confessed that the answer does not appear any clearer now that the problem is before us at close quarters than it did when it was surrounded by a halo of distance and of doubt. The statutes and regulations made by the Commissioners appointed under the University of London Act of 1898 have been passed by the House of Commons, and already the foundation-stone of a new hall for the work of one new faculty, that of Economics and Political Science, has been founded. The teaching of medical subjects is, however, a much more complex affair. It is scattered over many subjects and many places, and what seems sufficiently clear is that unless some concentration of the teaching in certain subjects can be effected the reconstituted University will remain as it was before, so far as its medical faculty is concerned, a mere degree-giving institution. Yet, in regard to a subject in which science and its practical application are so intimately commingled as is the case in medicine, concentration of teaching is hedged round with peculiar difficulties. If one started with a clean slate, if there were no vested interests, and if the large amount of money already sunk in school buildings would not be thrown on the hands of those responsible for them by any scheme for divorcing science teaching from hospital practice, and concentrating it in and around the new home of the University, the problem would be much simplified. But, taking things as they are, it seems impossible to avoid the conclusion that if concentration is to be effected it will have to be at the expense of the smaller and less perfectly equipped schools, and it seems equally impossible to avoid the conclusion that much heart-burning will be the consequence of any such attempt. If some rich man would but come forward and endow a great University school in which pure science could be taught, and in which the would-be medical student could complete all his earlier and intermediate studies in absolute independence of all connection with the hospital, where his purely medical studies would be undertaken, then the University would but have to "sit tight" and offer good teaching well fitted for the standard set up by its examinations. Before long students would flock to its lecture halls; the hospital schools would cease to trouble about many of the subjects by the teaching of which they are at present much hampered, and all would be well. London would become, as it ought to become, a great school of medicine; the University would itself teach the sciences on which the art of medicine is founded, and each of the hospitals would become a subsidiary college, at which the practical part of the curriculum would be gone through. But the rich man has not yet declared his whereabouts, and it is clearly impossible for an impecunious University to compete with schools, some of which are already in a high state of efficiency, although perhaps not exactly the sort of efficiency which we should look for in so great a medical centre. The only form, then, in which concentration seems at all possible lies in the proposal which has been made that the University should create, in connection with some of the larger of the existing medical schools, University institutes in which the intermediate subjects should be taught, hiring the existing buildings for the purpose. These institutes would then, taken altogether, constitute the University school in anatomy, or physiology, or such other subject as might be taught therein, and much of the difficulty arising from the hostility and competition of the larger schools would be met. Unfortunately, it would be met at the expense of sacrificing the interest of the smaller ones, for, whatever may be said to the contrary, no one can believe for a moment that a school which has to send its students out for their University courses will ever compete on equal terms with one which can give within its walls every course which is required for all examinations. This, then, although perhaps the best that offers, is not an altogether happy way out of the difficulty, for we cannot but feel that such a division of the teaching institutions into "University schools" and "mere College and Hall schools" will not only do injury to the hospitals to which the latter are attached, but will increase the gap which already exists to an injurious degree between the "University" and the "mere College and Hall" students. The best plan would be the concentration of the University teaching of the early and intermediate subjects at a great central well-endowed University School in Science, and the creation of purely medical colleges in connection with hospitals, or perhaps in some cases with groups of hospitals including some of those devoted to special subjects. But this cannot be unless the great central science school is well endowed. Let us pray, then, for a millionaire—a man sufficiently wealthy, sufficiently enlightened, and sufficiently anxious for undying fame to give us the school we want, and thus to hand his name down to posterity as one of the greatest benefactors of the greatest city in the world.

### Immunity.

In view of all that has been and is being done among civilised nations in the direction of sanitary improvement, and of the obvious diminution in the mortality from certain "filth diseases" which has resulted therefrom, it is not without interest to inquire how far such efforts are likely to improve our position as a colonising and a conquering race. The controlling influences which put a limit to the spread of infectious diseases

ANNOTATIONS—*continued.*

(and the word infectious now covers a far wider field than our fathers even thought it would) are, on the one hand, those which lead to the elimination of the micro-organisms which are regarded as the germs of these diseases; and, on the other hand, the development among the people exposed to them of some degree of "immunity" or "resistance" to their attacks. There is much reason for believing that by prolonged exposure to the attacks of disease germs for generation after generation, or, as in the case of yellow fever, from birth onwards, man becomes "salted," and develops a certain degree of immunity to their evil effects. Perhaps his tissues learn to resist them so that they can no longer grow in him; or, far more likely, he becomes indifferent to their presence. We have come to know that many apparently healthy people carry about with them the germs of diphtheria. They have become immune to diphtheria, probably by having undergone repeated small infections; and the same thing is known to happen in regard to typhoid fever. While large numbers of our soldiers are dying every year in India from typhoid fever, many of the natives who are living amid infinitely worse conditions yet escape, and it may fairly be asked, Do not the Hindus escape because they have become immune? and do not we suffer because with the lessening prevalence of typhoid in England we have lost our immunity? This question becomes one of very great importance in view of the possibility of our undertaking extensive military operations in China, a country the people of which live and seem to thrive in the midst of filth so indescribable that according to every sanitary theory it ought to decimate if not entirely to wipe out all who are exposed to it. Much has been said about the filth of the Boers, and it would be interesting to know how far they have suffered from filth diseases. It is openly asserted that they have not had much sickness. Have they then been entrenched behind an immunity bred of familiarity? The same about the Chinese. Is it because they have become immune that they can live and thrive and multiply and possess the land in defiance of the insanitary surroundings such as ought to kill a more nicely brought up European? and is the filthiness of their towns to be their protection against invasion by a more cleanly race? That it will be one of their strongest weapons of defence we cannot doubt.

### Chrome Poisoning.

Among the various subjects dealt with in the recently issued annual report of the Chief Inspector of Factories and Workshops, many of which serve to give distressing proof of the dangers to which our industrial army is constantly exposed, one of the most interesting is that on the influence of chrome compounds upon the health of those working among them, a subject which is treated at considerable length by Dr. T. M. Legge, medical inspector. Certainly, from the account given of the injuries produced by these compounds, there is ample reason for the imposition of the amended "special rules" which have now been issued. The irritating dust which arises in chrome works seems to be the chief cause of the trouble. It is, in fact, a caustic, which when introduced into any scratch or wound sets up ulceration of a curiously obstinate character. When the resulting ulcers occur on the skin they are spoken of as "chrome holes," but often they take place on the septum of the nose, which they almost inevitably perforate, this perforation being the particular morbid state which may be regarded as special to this industry, and although neither of these conditions may be a menace to life, they are of quite sufficient gravity to make it extremely important that every care should be taken to prevent their occurrence. The point which is attacked in the nose is a point about a quarter of an inch from the lower and anterior margin of the septum, and the ulceration extends upwards until it reaches the junction of the septum with the ethmoid and backwards to the vomer. When it has reached these limits healing takes place, but the cicatrix generally becomes covered with an echthymatous crust of mucus. The chrome holes are sluggish and often sloughing ulcers, caused by a cut or abrasion of the skin coming in contact with crystals or solution of bichromate. Truly such accidents are distressing enough, and their occurrence serves to emphasise very strongly the necessity for such inspection and supervision of industries as is afforded by the Home Office.

### Small-pox.

The interesting little outbreak of small-pox which has recently taken place in and around Kensington serves at least to teach us that this disease, however thoroughly it may be suppressed by vaccination, and kept in check by isolation, is but seldom far away, and is liable at any moment to become epidemic if the opportunity be offered. The special lesson, however, which is taught by this particular outbreak is as to the care which it is necessary to exercise in the diagnosis of those irregular or abortive forms of eruption which are apt to characterise the disease when it is modified by a partially protective vaccination. In the Kensington cases, the three diseases which appear to have been simulated were measles, chicken-pox, and eczema. The mistake between small-pox and measles is not uncommon, and is often quite excusable. Whenever the former disease is prevalent a few cases occur in which the on-coming of measles is mistaken for that of small-pox; while the initial rashes of small-pox, and even the eruption itself on the first or second day of the confluent form, are not infrequently mistaken for the rash of measles. The great imitator of small-pox, however, is chicken-pox. Yet the differences between the two are very marked, especially if one can make sure of the history, and can see the eruption before it has been deformed by scratching; but with a fading eruption, and with the careless history given by an unobservant mother, it is to be admitted that serious difficulty sometimes arises. In 1895, the last year in which there has been any serious prevalence of small-pox, 63 cases removed by the ambulances of the Metropolitan Asylums Board, and certified as suffering from small-pox, were found on arriving at the wharves to be affected with chicken-pox only. Turning now to the year 1898, in which the total number of small-pox cases admitted by the Metropolitan Asylums Board amounted to only five, we find that 31 cases were sent in certified as small-pox, and that in 17 of these chicken-pox turned out to be the real malady. It is clear that, as the result of the careful manner in which small-pox has been dealt with by the authorities during the past 20 years, a large number of medical men have seen so little of it that they find considerable difficulty in diagnosing the disease.

